# Role of Hypoxic Stress in Regulating Tumor Immunogenicity, Resistance and Plasticity

**DOI:** 10.3390/ijms19103044

**Published:** 2018-10-06

**Authors:** Stéphane Terry, Rania Faouzi Zaarour, Goutham Hassan Venkatesh, Amirtharaj Francis, Walid El-Sayed, Stéphanie Buart, Pamela Bravo, Jérome Thiery, Salem Chouaib

**Affiliations:** 1 INSERM UMR 1186, Integrative Tumor Immunology and Genetic Oncology, Gustave Roussy, EPHE, Fac. de médecine—Univ. Paris-Sud, University Paris-Saclay, Villejuif F-94805, France; stephanie.buart@gustaveroussy.fr (S.B.); pamelisa.b@gmail.com (P.B.); 2 Thumbay Research Institute of Precision Medicine, Gulf Medical University, Ajman 4184, United Arab Emirates; dr.rania@gmu.ac.ae (R.F.Z.); dr.goutham@gmu.ac.ae (G.H.V.); francis@gmu.ac.ae (A.F.); walidshaaban@gmu.ac.ae (W.E.-S.)

**Keywords:** hypoxia, tumor microenvironment, tumor heterogeneity, cancer, cancer stem cells, EMT, cell plasticity, DNA damage and repair, immune evasion, HIF

## Abstract

Hypoxia, or gradients of hypoxia, occurs in most growing solid tumors and may result in pleotropic effects contributing significantly to tumor aggressiveness and therapy resistance. Indeed, the generated hypoxic stress has a strong impact on tumor cell biology. For example, it may contribute to increasing tumor heterogeneity, help cells gain new functional properties and/or select certain cell subpopulations, facilitating the emergence of therapeutic resistant cancer clones, including cancer stem cells coincident with tumor relapse and progression. It controls tumor immunogenicity, immune plasticity, and promotes the differentiation and expansion of immune-suppressive stromal cells. In this context, manipulation of the hypoxic microenvironment may be considered for preventing or reverting the malignant transformation. Here, we review the current knowledge on how hypoxic stress in tumor microenvironments impacts on tumor heterogeneity, plasticity and resistance, with a special interest in the impact on immune resistance and tumor immunogenicity.

## 1. Introduction

The tumor microenvironment (TME) is a complex system that consists of the extracellular matrix (ECM) and numerous cell types including fibroblasts, adipose cells, immune cells, endothelial cells as well as components of the blood and lymphatic vascular networks and the nervous system. TME plays an important role in tumor development and progression [1,2,3]. It involves soluble factors and metabolic changes. Among these metabolic changes, hypoxia plays a pivotal role in shaping the TME [4,5]. In such a system, hypoxia appears as an essential metabolic element to control cellular plasticity and tumor heterogeneity [6,7]. It is well established that hypoxic stress is a feature of most solid tumors and is associated with poor clinical outcomes in various cancer types [2,8,9,10,11]. Hypoxia arises due to a combination of excessive oxygen consumption by growing tumor cells and the disorganized tumor-associated vasculature [3]. Considerable evidence now suggests that hypoxia plays an important role in tumor progression, affecting both metastatic spread and selection of cells with more aggressive phenotypes [7,12,13]). This is at least partly explained by the fact that hypoxia can promote cancer cell stemness, invasion or metastatic capacities via the activation of hypoxic cascades and hypoxia-inducible factors (HIFs). To date, the mechanisms at play are still far from being understood. The adaptive responses to hypoxia are regulated by HIFs. The master regulator of the hypoxic response is the hypoxia-inducible factor 1 (HIF-1). In mammalian cells, the response to hypoxia depends in large part on the activation of HIF-1, a heterodimeric transcription factor consisting of a hypoxia-inducible HIF-1α subunit and a constitutively expressed HIF-1β subunit [14]. HIF-1 transactivates target genes containing *cis* acting hypoxia response elements that contain the HIF-1-binding site sequence. HIF-1α protein levels are tightly regulated by the cellular pO2. Under hypoxic stress, hypoxia-dependent stabilization of HIF dimers allows for the induction of numerous genes regulating various biological processes and functions in cells, including angiogenesis, cell survival, proliferation, pH regulation, and metabolism [4].

## 2. Hypoxia Induced Tumor Plasticity and Heterogeneity

Tumors contain distinct cell types that collectively create microenvironmental conditions controlling the tumor growth and its evolution. Insufficient concentration of oxygen in the growing tumor generates hypoxic stress, which can lead to metabolic, epigenetics and phenotypic reprogramming of the cells coincident with fluctuations in the composition of the microenvironment [15,16], while potentially affecting the functions, the phenotype and/or the number of microenvironmental cell components [5,6]. As a corollary, hypoxia should be considered as a driver of cell plasticity, since it can promote the capacity of a cell to shift from its original cellular state to a distinct cellular state. One interesting unanswered question is the impact of hypoxic stress on tumor heterogeneity. It is well established that tumors exhibit substantial heterogeneity with potential consequences on their evolution in time and response to treatments [17,18,19,20]. So far, the extent of this heterogeneity has been only partially explored, especially in relation to the diverse mutational landscapes found in tumors [17]. Clearly, more work is now needed to explore and define the phenotypic heterogeneity of the various cell types. The advent of single-cell approaches offers a unique opportunity to gain insights into tumor heterogeneity [21,22,23,24]. Recently, using breast tumors, Azizi and colleagues nicely showed that environmental factors, including hypoxia present in the tumor, but marginal in the normal tissue, were linked to the increased diversity of immune phenotypic states of T cells, myeloid cells and Natural killer (NK) cells [23]. Tumor-resident T cells appeared to be particularly responsive to such regulation, as shown by the increased number of gene signatures activated in highly hypoxic tumors. The findings also suggest that various degrees of hypoxia, inflammation, and nutrient supply, or a combination of these factors in the local microenvironment could lead to a spectrum of phenotypic states while promoting the enrichment of certain subpopulations such as the Treg subset. The work of Palazon et al. recently revealed the essential role of HIF-1α in regulating the effector state of CD8+ T cells [25]. Hypoxia stimulated the production of the cytolytic molecule granzyme B in a HIF-1α- but not HIF-2α-dependent fashion. Importantly, hypoxia through HIF-1α also increased the expression of activation-related costimulatory molecules CD137, OX40, and GITR, and checkpoint receptors PD-1, TIM3, and LAG3. This may have important implications for tumor immunology. Further experimental data from these investigators already denote the importance of the HIF1/VEGF-A axis to promote vascularization and T cell infiltration.

**Figure 1:**
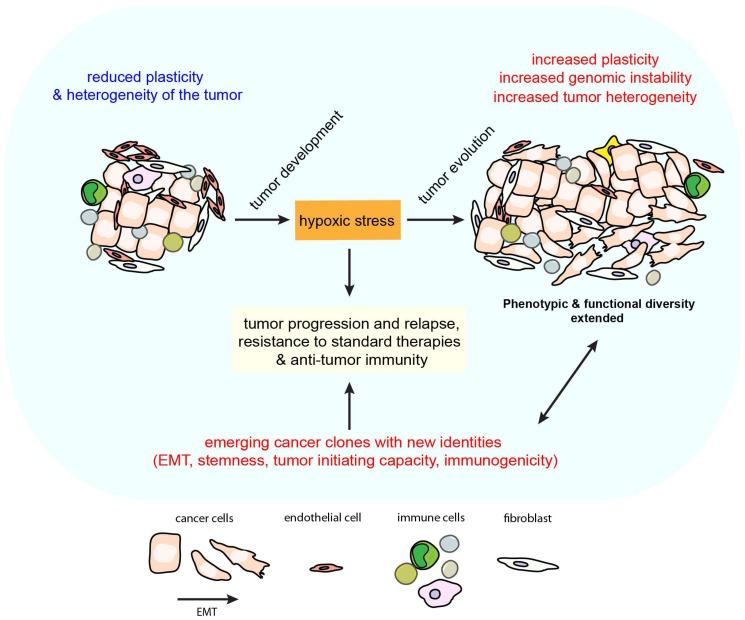
Tumors contain distinct cell types that collectively enable tumor growth and progression. Hypoxic stress can contribute by increasing cell plasticity, genomic instability and phenotypic heterogeneity of certain carcinoma cells, leading to intra-tumor heterogeneity and the emergence of cancer clones resistant to therapies and anti-tumor immunity.

Aside from its impact on stromal components, the cell plasticity of cancer cells represents a major source of phenotypic heterogeneity in the tumor. Here again, HIFs, angiogenesis and inflammatory factors such as VEGF, or TGF-β (induced and activated under hypoxic conditions), might exert important regulatory functions. A prime example of this notion comes from the numerous studies demonstrating that all these factors can stimulate epithelial-mesenchymal transition (EMT) and/or support a mesenchymal state [13,26,27]. It is also well established that certain cancer cells have the capacity to transit between epithelial and mesenchymal phenotypes, or states, via epithelial-mesenchymal transition (EMT), or the reverse process, mesenchymal-epithelial transition (MET) [26]. In such a scenario, cancer cell plasticity is tightly regulated by signals perceived from the TME and anatomic sites. Notably, hypoxic stress might enable other types of phenotypic changes. For instance, HIF-1α and hypoxia could contribute to the neuroendocrine transformation of prostate tumors and adenocarcinoma cells through cooperation with the transcription FoxA2, reduced Notch-mediated signaling, and induction of neuronal and neuroendocrine gene programs in the cells [28,29,30]. Despite substantial evidence for a role of hypoxia in triggering EMT programs, the exact mechanisms at play remain relatively unclear. Both promoting and suppressing roles of hypoxia have been described in human and in mouse laboratory models [31,32,33,34,35]. In fact, our knowledge of what really occurs in patient tumors is still fragmentary. In this regard, the study of Puram et al. is particularly valuable [36]. These investigators profiled transcriptomes of ~6000 single cells from 18 head and neck squamous cell carcinomas. This included the analysis of 2216 malignant cells allowing the study of intra-tumoral phenotypic diversity of the cells. They found that malignant cells varied within and between tumors in their expression of signatures related to cell cycle, stress, hypoxia, epithelial differentiation, and partial EMT. One notable aspect of the findings was the strong correlation found between hypoxia and EMT signatures in the individual tumors. Similarly, we recently explored the relationship between hypoxia status and EMT-TF expression levels by analyzing lung adenocarcinomas included in the TCGA-LUAD project [37]. In this large cohort, hypoxia signatures, as well as HIF1A mRNA expression, were significantly and positively correlated with EMT-TF expression levels. In an attempt to better model the impact of hypoxia in non-small cell lung cancer (NSCLC), we exploited the primary NSCLC IGR-Heu cells and observed that EMT-related phenotypic changes were particularly exacerbated when hypoxic stress was maintained for a prolonged period. Moreover, under these experimental conditions, the shift towards a mesenchymal phenotype was only observed in a fraction of stressed cells. While some cells undergo EMT, others do not shift towards the EMT spectrum. Therefore, despite long-term exposure to hypoxic stress, a high proportion of clones retained epithelial features contributing to expand the phenotypic diversity in the cancer cell population (Figure 1) [37]. It is also interesting to keep in mind that in vivo, cancer cells may be exposed to chronic or intermittent hypoxic stresses, and depending on their location, to various hypoxia levels [38]. The propensity of hypoxic stress to generate cancer cell heterogeneity was further illustrated by the recent study of Lehmann and colleagues [39]. In their attempt to dissect how plasticity of tumor cell migration and EMT is involved in the early metastatic steps, they identified the hypoxia/HIF-1 axis as an inducer of amoeboid detachment and the production of heterogeneous cell subsets whose phenotype and migration were dependent or independent of Twist-mediated EMT. Taken together, these reports underscore the importance of hypoxic stress in mediating tumor plasticity and heterogeneity. 

## 3. Impact of Plasticity and Heterogeneity on Tumor Immune Escape

Evidence is accumulating that tumor plasticity and heterogeneity might be key determinants in the emergence of therapy resistant cancer clones (Figure 1) [19,40]. Considering the relationship between tumors and the immune system, it becomes quite clear that EMT or dedifferentiation can turn even highly immunogenic cancer clones into poorly immunogenic cancer variants resistant to T cell immune attacks through various mechanisms accompanying their phenotypic reprogramming [41,42,43]. This includes defects in the antigen-presentation machinery involving major histocompatibility complex (MHC) class I molecules, defects in immune recognition following loss of adhesion molecules, gain or loss of immune-modulatory factors and secretion of immunosuppressive substances, or gain of anti-apoptotic properties by the cancer cells against cytotoxic immune effectors. Thus, the acquisition of a more mesenchymal phenotype by cancer cells has been associated with deficiencies in the MHC I antigen presentation pathway [44,45,46,47], downregulation of E-Cadherin [37], which could be critical for the recognition of cancer cells by tumor infiltrating lymphocytes (TILs) expressing [48,49], hyperactivity of TGF-beta signaling [45], or increased expression of programmed death-ligand 1 (PD-L1) [46,50,51]. Such immune resistant phenotypes are not only relevant for resistance to T-cell-mediated killing. Numerous reports showed evidence of a link between acquisition of mesenchymal features by cancer cells and their relative protection from NK-cell-mediated lysis [37,52,53], or phagocytosis through direct or indirect mechanisms [54]. Ricciardi and colleagues observed that exposing carcinoma cells to inflammatory cytokines not only promotes EMT in these cells but also confers a number of immunomodulatory properties, including interference with proliferation, differentiation and apoptosis of NK, T and B cell populations [55]. On the other hand, immune cells such as macrophages and NK cells can also mediate EMT of cancer cells, and presumably, could influence immune resistant states of carcinoma cells [56,57,58].

A study by Huergo-Zapico et al. recently showed that NK-cells could mediate EMT programs in melanoma cells, simultaneously potentiating the immune resistance capacity of the latter. On the contrary, data from at least two studies using various model systems have raised the possibility that EMT induction could increase cancer cell susceptibility to NK cells through up-regulation of NKG2D ligands or cell adhesion molecule 1 (CADM1) [59,60]. This further highlights the contextual nature of the events. For a better understanding of these discrepancies, we suggest that special attention should be paid to the dynamic and the continuum of EMT states, as well as on the timing and the nature of the EMT inducers used to manipulate laboratory models. Considering the role of hypoxic stress in our recent study, we demonstrated that a prolonged hypoxic stress (1% O_2_) promotes EMT in the NSCLC IGR-Heu population in a manner that depends on the hypoxia effector HIF-1-α [37]. As mentioned above, while some cells experienced profound phenotypic changes toward mesenchymal states, others do not, thus generating cancer cell heterogeneity in the cancer cell population. This was reflected by the presence of a mixture of cells moving along the EMT spectrum with more epithelial or mesenchymal phenotypes. Among the cancer subclones emerging from this hypoxic stress, those with a more mesenchymal phenotype had an increased propensity to resist attacks by cytotoxic lymphocytes as compared to the more epithelial counterpart. This was illustrated by their reduced susceptibility to both cytotoxic T cells (CTL) and NK cell-mediated lysis [37]. In another study, hypoxia-induced EMT of hepatocellular carcinoma cells promoted an immunosuppressive TME by stimulating expression of indoleamine 2,3-dioxygenase (IDO) in monocyte-derived macrophages [61].

Work by Zhang indicates that HIF-1α can stimulate CD47 expression, an important factor for maintaining plasticity of the cells, which also enables breast cancer cells to avoid phagocytosis by macrophages [62]. CD47 hampers the “eat me signal” on cancer cells by interacting with SIRP on macrophages impairing phagocytosis. More recently, Noman and colleagues identified CD47 as a direct target of SNAI1 and ZEB1 [54]. They observed that the CD47 blockade sensitized cancer cells to phagocytosis, particularly in breast cancer cells with Mesenchymal features. In Triple-negative breast cancers (TNBCs), a heterogeneous group of breast tumors that can present many of the salient features found during EMT, the recent report by Samanta et al. revealed that several immuno-modulatory molecules including CD47, PD-L1 and CD73 are direct HIF target genes in TNBC cells [63]. Thus, CD47 expression could reduce killing by macrophages, whereas CD73 and PD-L1 mediate independent mechanisms to inhibit the T-cell effector functions. The coordinate transcriptional induction of these factors was especially observed in cells exposed to certain chemotherapeutic agents such as carboplatin, doxorubicin, gemcitabine, or paclitaxel. Taken together, this data gives great insight into how plasticity of the cancer cells can be linked to a multi-resistant phenotype involving resistance to chemotherapy and immune resistance. The high amount of TGF-β (another HIF target gene) produced by certain carcinoma cells, or the stromal compartment, could also be crucial in dampening the immune response in tumors [20,64,65,66,67]. Moreover, interactions between the different contingents should be highlighted. For instance, carcinoma cells with a mesenchymal or a partial EMT features could cooperate and interact with cancer associated-fibroblasts to regulate their phenotype, and presumably immune suppression [36]. Substantial evidence also indicates the role of HIF-mediated immune plasticity in shaping anti-tumor immunity [5,6,68]. As mentioned above, HIF1 could be a major regulator of effector CD8+ T cell functions [25]. An interesting study by Hatfield and colleagues reported that hypoxia reversal via supplemental oxygenation had significant anti-tumor effects in mouse models, resulting in long-term survival of the mice [69]. Importantly, the observed effects were mainly attributed to the presence of T and natural killer cells. Investigators further showed an association with increased intratumoral infiltration, reduced immunosuppression by regulatory T cells and inhibition of tumor-reactive CD8 T cells concomitant with increased pro-inflammatory cytokines and decreased immunosuppressive substances including TGF-β. It is known that dendritic cell differentiation and maturation is impaired under hypoxia, with negative effects on their T-cell activating functions [70]. The work of Facciabene and colleagues invoked the role hypoxia in the recruitment of Tregs through inducing expression of chemokine CC-chemokine ligand 28 (CCL28), which in turn, promotes angiogenesis and tumor tolerance [71]. Further research also indicates the direct role of HIF-1α in regulating the functionality and plasticity of T-regs [72,73]. Myeloid-derived suppressor cells (MDSCs) and tumor-associated macrophages (TAMs) are also known to contribute to tumor-mediated immune escape [74]. Eubank and colleagues showed evidence for a role of HIF-1 and HIF-2 in the promotion of macrophage angiogenic property [75]. HIF-1α could also regulate their inhibitory functions on T cells [4,5]. Interestingly, the study of Corzo et al. showed that hypoxia via HIF-1α could somehow extend the suppressive function of tumor MDSCs while redirecting their differentiation toward macrophages in the TME [76]. Finally, we showed that hypoxia could regulate the tumor MDSC functions by direct transcriptional induction of the programmed death-ligand 1 (PD-L1) in these cells, resulting in increased MDSC-mediated T cell tolerance [77].

## 4. Mechanisms of Hypoxia-Induced Cancer Stem Cells

CSCs are a subpopulation of cancer cells that have the ability to self-renew, to divide, to give rise to another malignant stem cell and to drive tumor growth and heterogeneity [78,79]. Hypoxia is a significant culprit for the development of tumor cell resistance to therapy, which is in part due to the generation of cancer stem cells (CSCs) [80,81,82]. Both HIF-1α and HIF-2α have been found to contribute to the mechanisms involved in mediating stemness [80,82,83,84]. Despite numerous studies in cancer model systems, the molecular mechanisms underlying the CSC generation, downstream of HIFs have not yet been completely elucidated. So far, they have been explored in various cancer models. HIF proteins can directly or indirectly regulate the expression of genes involved in the initiation and maintenance of stem cells such as (OCT4, SOX2, KLF4, MYC, NANOG, CRIPTO, Wnt or NOTCH) [80,85,86,87,88]. In addition to their essential functions during embryonic development, these genes could exert diverse functions in cancer. In certain human tumors, they might represent valuable tools to predict recurrence and tumor plasticity, although such prognostic value is far from being established [79,89,90,91,92,93,94].

In response to hypoxia, HIF-2α was shown to upregulate OCT4 and SOX2 expression resulting in an increase in the migratory capacity of glioma cells [95,96]. In the study of Tang et al., increased levels of HIF-1α in colorectal cancer cells was associated with increased chemoresistance through the GLI2 transcription factor, which coincides with an increase in cancer stem cells [97]. Similarly, HIF-1α and HIF-2α have been shown to increase the expression of the stem cell marker CD133 in glioblastoma cells concurrent with increased chemoresistance [98]. In breast cancer cells, HIF-1α and HIF-2α increased NANOG mRNA by stimulating expression of AlkB homolog 5 (ALKBH5), an m(6)A demethylase able to demethylate NANOG mRNA [99]. HIF-1 was required for the activation of the p38 MAP kinase pathway and inhibition of ERK signaling resulting in stabilization of NANOG, KLF4, and enrichment of breast cancer stem cells [100]. Clearly, understanding how these different signaling mechanisms interact to drive tumor progression and therapy resistance under variable oxygenation conditions will be critical to the efforts to develop more effective cancer therapies.

## 5. EMT at the Crossroad of Stemness

EMT has been proposed to drive invasion, resistance to therapy, and spreading of cancer to distant sites [13,26,27]. Cells that are committed to EMT also exhibit numerous attributes that are known to be characteristics of stemness [101]. Although cancer stem cells account for only a small part of the tumor bulk, they are assumed to be the main players involved in therapeutic resistance, cancer relapse, and distant metastasis. Hypoxia and HIF proteins likely contribute to the molecular link between EMT and stemness (Figure 1). Indeed, HIFs are not only involved in the regulation of stem cell factors, in response to hypoxic stress HIF1 protein activates the expression of EMT-transcription factors TWIST1 or ZEB1, which ultimately promotes EMT [31,102]. HIF1 can also help cells transition to a more mesenchymal phenotype by regulating the lysyl oxidases LOX and LOXL2, leading to repression of E-cadherin [34,103]. Other studies have reported that Notch signaling and the EMT-TF SNAIL could be involved in this network as well [35]. It is important to note that cancer cells undergoing EMT in response to hypoxia will not only gain mesenchymal properties, but also may acquire stem cell-like features [104]. Signaling pathways leading to EMT involves TGF-β, STAT3, miR-210 among others (Notch, Nanog) [26,104,105,106]. TGF-β expression is regulated by HIF-1, and in turn, TGF-β plays an important role in stabilizing HIF-1 [37,107]. TGF-β has been described as having a dual function both in suppressing as well as promoting cancer stem cell populations [108]. The effect of TGF-β has also been correlated with the stage of the cancer; at early stages TGF-β has anti-growth effects, whereas at late stages, it promotes the development of aggressive growth [109]. Interestingly, in breast cancer, stem cell-like cells obtained after TGF-β exposure showed resistance to radiation therapy [110]. Likewise, renal cell carcinoma cells having acquired a stem-like phenotype after TGF-β-induced EMT showed an increase in chemoresistance [111]. In gynecologic cancer patients, the use of chemotherapy can induce TGF-β signaling resulting in reduced chemosensitivity [112]. In primary lung cancer cells, TGF-β exposure led to an increase in cancer stem cell population through repression of miRNA138 [113] while in colon cancer, TGF-β seems to play a key role in angiogenesis, tumor growth and metastasis [114]. On the other hand, in the context of hepatocellular carcinoma, TGF-β resulted in a decrease in cell survival of stem-like side populations [115]. Collectively, these studies highlight the importance of HIFs and TGF-β in the regulation of EMT, and provide support for the development of strategies exploiting these pathways to overcome therapy resistance.

It should be noted that STAT3 also plays an important role in the regulation of cancer stem cells and therapy resistance [116,117]. STAT3 has been demonstrated to be a potent stabilizer of HIF-1 in multiple cancer cell models [118,119,120,121]. Moreover, at the molecular level, STAT3 signaling is complex and cooperates with several other pathways implicated in cancer growth. This has recently been reviewed by Galoczova et al. [116]. There is currently a need and ample room to better explore STAT3 implications in EMT, cancer stem cells and tumor resistance to therapy. Of particular interest is the development of strategies for STAT3 inhibition, which has been shown to induce apoptotic cell death of STAT3 dependent cancer cells [122]. MicroRNAs also deserve particular attention. They are small non-coding RNAs that function in post-transcriptional regulation of gene expression and in mRNA silencing. Recent studies unraveled the role of hypoxia in the regulation of microRNA machinery components Drosha and Dicer in cancer cells with important consequences for miRNA biogenesis and tumor progression [123,124]. In particular, this work points to the role of hypoxia in promoting EMT and stem cell phenotypes through mechanisms involving oxygen-dependent H3K27me3 demethylases KDM6A/B or HIF-1 target ETS1/ELK1, which ultimately may lead to derepression of certain EMT-TFs such as the miR-200 target ZEB1. On the other hand, miR-210 is highly induced in response to hypoxic stress and it regulates HIF expression [125,126]. miR-210 is known to have important functions during cancer progression, with both promoting and suppressive roles [127]. Inhibition of miR-210 through small molecules results in inhibition of tumorigenesis in a mouse model for triple negative breast cancer [128]. In ovarian cancer cells, it was found to be a promoter of EMT by causing a decrease in E-cadherin, and increase in vimentin [129]. In lung cancer cells, miR-210 was found to regulate the susceptibility of cancer cells to lysis by cytotoxic T cells [126].

## 6. Hypoxia, DNA Repair and Genomic Instability

Hypoxic regions are heterogeneous within tumors and the hypoxic phase can vary with time and intensity (acute and/or chronic). Accumulating evidence demonstrates that this component of the TME can be associated with an increase in genomic instability of tumor cells, covering a wide range of alterations, from point mutations to chromosomal instability. The magnitude of genetic aberrations such as increases in gene mutations and gene amplifications due to variation in severity of hypoxia can be 5–1000-fold [130]. Indeed, several studies have suggested that hypoxia can induce DNA damage, alter cell cycle checkpoints and/or the sensing and repair of DNA damage, and consequently favor genetic instability (Figure 1) [131,132,133]. In this regard, several teams have documented an increase in the rate of DNA mutations in cells exposed to in vitro or in vivo hypoxic conditions, mostly using reporter assays [134,135,136]. The origins of these hypoxia-induced DNA mutations are probably multiple, emerging from hypoxia-mediated oncogene amplification, induction of DNA damages or DNA replication stress, deregulation of DNA damage checkpoint signaling, interference with DNA repair or escape from cell death [132]. Importantly, cycles of hypoxia and re-oxygenation are common phenomena seen in solid tumors and characterized by an increase in the intracellular free radical species [133,137,138], which are also strongly associated with accumulation of DNA damage [133]. However, in the absence of re-oxygenation (chronic hypoxia), hypoxia-induced genetic instability mostly arises from the influence of hypoxic conditions on DNA-damage repair pathways or the induction of a replicative stress, without detectable induced DNA damage [131]. It will be important to further investigate this intriguing possibility, especially in vivo.

In the case of DNA damage, the G1/S and the G2/M checkpoints kinases ataxia telangiectasia mutated [139], ATM-Rad3-related (ATR) and CHK2/CHK1, respectively, transmit signals to the effector molecules such as p53, p21 and CDC25 to prevent cell cycle progression or to initiate programmed cell death. Interestingly, emerging evidence suggests that different severities and durations of hypoxia may have different effects on cell cycle checkpoint controls. For example, oxygen levels such as 0.2% can bypass ATM or ATR and cell cycle checkpoint signaling allowing the propagation of tumor cells with potentially altered DNA that can contribute to genomic instability [140]. Furthermore, it has been proposed that hypoxia can exert selective pressure that leads to expansion of tumor cells with reduced apoptotic capacity due to, for example, TP53 mutations [141], which is considered as the guardian of genome integrity. As mentioned above, DNA repair pathways, especially homologous recombination (HR), mismatch repair (MMR), non-homologous end joining (NHEJ) and base-excision repair (BER) have also been shown to be compromised under hypoxic conditions [131,132]. For example, it was demonstrated that hypoxia can decrease the expression of the HR-related protein RAD51 in a HIF-1α independent manner [142]. Similarly, reduced expression of the NHEJ-related proteins DNA-PKcs, Ku70, Ku80 and DNA-ligase IV has been observed in hypoxic conditions [143]. Hypoxia has been also shown to transcriptionally downregulate the MMR genes MLH1, MSH2 and MSH6 [144] and a hypoxia driven microsatellite instability (MSI) has been proposed [132]. This hypoxic modulation of DNA repair pathways is thus thought to be of major importance in the genomic instability induced by chronic hypoxic conditions.

The induction of DNA damage, the alteration of DNA-damage cell cycle checkpoints and a functional decrease in the DNA repair pathways under hypoxic conditions probably contribute to “mutator” phenotypes in hypoxic cells and to genomic instability, which might have important effects on the anti-tumor immune response and tumor immunogenicity. For example, recent studies have provided new insights into how specific genomic alterations deriving from genome instability can impact on immune evasion of antitumor immunity [43]. Moreover, recent findings demonstrate the role of double strand break repair pathway in up-regulation of PD-L1 expression by cancer cells [145]. However, the influence of hypoxia-induced DNA-damages in PD-L1 expression is currently unknown. Importantly, a potential mutational burden in hypoxic cells could also be linked to their immunogenicity. Indeed, during the past few years, several groups have identified cancer rejection antigens formed by peptides that are entirely absent from normal human tissues, so-called “neo-antigens”. Such neo-antigens are solely created by tumor-specific DNA alterations/mutations that result in the formation of novel protein sequences. As compared with non-mutated self-antigens, neo-antigens are thought to be of particular relevance to tumor control, as the quality of the T cell pool that is available for these antigens is not affected by central tolerance [146]. As a result, neo-antigens appear to represent ideal targets for T cell-based cancer immunotherapy [147]. In this regard, tumors harboring deleterious mutations in the DNA repair pathways were found to carry a high number of candidate neo-antigens, which is associated with a clinical benefit from immune checkpoint inhibitor therapy (anti-PD1) (Le, 2015 #2213), indicating that a high burden of tumor neo-antigens correlates with a durable response to anti-immune checkpoint-based immunotherapy. Two recent studies revealed that mutations and/or loss of the DNA repair mechanism leads to increased mutational load, thus resulting in enhanced neo-antigen generation in cancer cells [148,149]). Nevertheless, the hypothesis that hypoxia-induced DNA damages/genomic instability can lead to a high mutational burden and high numbers of neo-antigens, increasing the potential immunogenicity of hypoxic cells, has never been validated.

## 7. Therapeutic Targeting of Hypoxia in Cancer

In view of the importance of the link between cancer stem cells, cell plasticity and therapeutic implications in cancer development, targeting the hypoxic niches may offer a great advantage in anti-cancer therapy. This is because targeting hypoxic niches results in eliminating diverse cell populations including cancer stem cells, and preventing the commitment of certain highly plastic cells to an EMT program [27,150].

In support of this idea, it was shown that oxygen administration to patients does transiently relieve tumor hypoxia, and as a result, improve therapy [151]. As such, detection of hypoxic areas in vivo is an essential first step. Recent development of two-photon molecular probes in detecting hypoxia in tissue in vivo and in vitro recently demonstrated some efficacy in detecting hypoxia in deep tumor tissue [152]. However, its effective use in the human situation needs to be established. Another important issue to be addressed is for the drugs to be targeted to the hypoxic zones. Several approaches for targeting hypoxic tumor cells are being explored including hypoxia-activated prodrugs, gene therapy, recombinant anaerobic bacteria and specific targeting of HIFs, or targeting pathways important in hypoxic cells such as the mTOR and UPR pathways [27,153,154]. Hypoxia activated prodrugs are drugs that are converted to their active state under a hypoxic environment. These have been developed and used in combination with chemotherapy or targeted therapy [155]. Recombinant anaerobic bacteria have been considered as gene delivery vehicles to cancer cell sites and spare normal tissue. The *Clostridium* strain that expresses prodrug-converting enzymes has been used, allowing for high therapeutic doses in the tumor [156]. A combination of hypoxia-activated drugs with nanotechnology can be used to enhance tumor specific delivery of anti-cancer agents to the hypoxic tumor zone.

HIF1, being presumably the most powerful factor in the hypoxic response represents an ideal target for therapy. In this regard, the development of selective HIF-1α antagonists remains an important clinical challenge [157,158]. Nonetheless, molecule inhibitory drugs reducing HIF-1α levels, or targeting HIF1 stability/activity may provide interesting benefits in anti-cancer therapy. Nanoparticle formulations containing amino bisphosphonate zoledronic have been successfully used in combination with doxorubicin to sensitize cancer cells to multidrug resistance through inhibiting HIF-1 [159,160]. Inhibitors affecting HIF protein translation include cardiac glycosides, PX-478 or topoisomerase I inhibitors [161,162,163,164,165,166,167]. Translation of HIF1 mRNA is known to be controlled by the PI3K/AKT/mTOR pathway. Inhibition of this pathway could decrease HIF expression and the resultant tumorigenesis [168,169,170]. As an alternative, targeting pathways downstream of HIF signaling includes the use of anti-VEGF therapy (monoclonal antibodies targeting VEGF (bevacizumab) or small molecule inhibitors targeting the VEGF receptor), which has been used for anti-angiogenic/vascular normalization effects in certain medical indications including ovarian, renal, lung or colorectal cancers, in combination with chemotherapy [171]. Finally, recent studies give promise to our ultimate ability to design specific inhibitors of HIFs. A new class of HIF antagonists are currently being tested and have already proven to selectively target HIF-2α with relatively low toxicity compared to current anti-angiogenic drugs [158,172,173,174].

## 8. Conclusions

Expansion of resistant cancer cell clones during cancer treatment is a major issue for cancer therapy. It reflects a clonal evolution resulting from genomic instability, cellular plasticity and activation of stemness pathways, as well as complex regulatory networks orchestrated by the TME. Tumor microenvironmental hypoxia is a relevant example that demonstrates how microenvironmental parameters can interfere and neutralize immune cell functions. Converging evidence now suggests its potential value as a prognostic factor as well as a predictive factor owing to its multiple contributions to chemoresistance, radio resistance, angiogenesis, resistance to cell death, altered metabolism, genomic instability, cell plasticity and various immune-related aspects. There is a clear rationale to develop efficient ways to target microenvironmental hypoxia to prevent tumor evolution and the emergence of therapy resistance. However, information on the mechanisms at play is still fragmentary and may vary in a contextual manner. Despite insightful experimental studies using in vitro or in vivo models, the challenge remains for scientists and clinicians alike to gain a better understanding of how human tumors respond to hypoxia. It will also be critical to develop specific agents for targeting hypoxia and associated pathways. This has the potential to provide innovative cancer therapies that can enhance antitumor immunity and overcome the barriers of treatment resistance, tumor tolerance and escape from immune surveillance. In the era of cancer immunotherapy, current strategies such as immune checkpoint blockade have focused on attempting to target immune cells directly to boost the immune system of the host. Is it possible to use therapeutic targets derived from the hypoxic TME and associated pathways as new therapeutic solutions for immunotherapy of cancer? This question merits further investigation. An important challenge will be to determine the best combination strategies as well as the optimal timing and sequence of these combinations.

We are still at the beginning of an exciting period of discovery, and integrating the manipulation of hypoxic stress in cancer immunotherapy may lead to more durable and effective cancer immunotherapy approaches in the future.
